# Hypersensitivity pneumonitis: a complex lung disease

**DOI:** 10.1186/s12948-017-0062-7

**Published:** 2017-03-07

**Authors:** Gian Galeazzo Riario Sforza, Androula Marinou

**Affiliations:** Division of Sub Acute Care, Sesto San Giovanni Hospital, ASST Nord Milano, Sesto San Giovanni, Italy

## Abstract

Hypersensitivity pneumonitis (HP), also called extrinsic allergic alveolitis, is a respiratory syndrome involving the lung parenchyma and specifically the alveoli, terminal bronchioli, and alveolar interstitium, due to a delayed allergic reaction. Such reaction is secondary to a repeated and prolonged inhalation of different types of organic dusts or other substances to which the patient is sensitized and hyper responsive, primarily consisting of organic dusts of animal or vegetable origin, more rarely from chemicals. The prevalence of HP is difficult to evaluate because of uncertainties in detection and misdiagnosis and lacking of widely accepted diagnostic criteria, and varies considerably depending on disease definition, diagnostic methods, exposure modalities, geographical conditions, agricultural and industrial practices, and host risk factors. HP can be caused by multiple agents that are present in work places and in the home, such as microbes, animal and plant proteins, organic and inorganic chemicals. The number of environment, settings and causative agents is increasing over time. From the clinical point of view HP can be divided in acute/subacute and chronic, depending on the intensity and frequency of exposure to causative antigens. The mainstay in managing HP is the avoidance of the causative antigen, though the complete removal is not always possible due to the difficulties to identify the agent or because its avoidance may lead to major changes in life style or occupational settings. HP is a complex syndrome that needs urgently for more stringent and selective diagnostic criteria and validation, including wider panels of IgG, and a closer collaboration with occupational physicians, as part of a multidisciplinary expertise.

## Background

Hypersensitivity pneumonitis (HP), also called extrinsic allergic alveolitis, is a respiratory syndrome involving the lung parenchyma and specifically the alveoli, terminal bronchioli, and alveolar interstitium, due to a delayed allergic reaction. Such reaction is secondary to a repeated and prolonged inhalation of different types of organic dusts or other substances to which the patient is sensitized and hyper responsive, primarily consisting of organic dusts of animal or vegetable origin, more rarely from chemicals [1]. In this condition, that was first described in 1713 by the Italian researcher Bernardino Ramazzini in subjects belonging to 52 different professions, a repeated exposure to particles sufficiently small (diameter < 5 μm) to reach the alveoli and to trigger an immune response is necessary. The most at risk professional categories are workers in environments or settings contaminated by organic dust of various origin, mostly farmers or breeders. A lot of substances may be linked to this condition such as avian dust, mold, paint catalyst, sugar cane dust, hay dust, mushrooms, rat or gerbil urine, tobacco, heating and cooling systems water, maple bark dust, redwood bark dust, beer brewing, cork dust, plastic residue, epoxy resin, enzyme detergents, wheat mold or dust. The syndrome is greatly variable in symptoms severity, clinical presentation and prognosis, depending on the nature of causative agent, the duration of exposure, the host factors and the characteristics of the antigen [2]. In most cases, HP can be reversed promptly identifying and removing the causative agent(s), which can be found in a lot of settings, including home, workplace, and recreational environments [3].

## Prevalence

The prevalence of HP is difficult to evaluate because of uncertainties in detection and misdiagnosis and lacking of widely accepted diagnostic criteria, and varies considerably depending on disease definition, diagnostic methods, exposure modalities, geographical conditions, agricultural and industrial practices, and host risk factors [2]. Moreover, HP develops only in a small part of individuals exposed to causing agents, while the majority of individuals exposed to the same agent are sensitized but asymptomatic or do not even become sensitized, suggesting the existence of a genetic predisposition [4]. To further complicate the issue, in some patients HP might be a reaction to a single environmental agent, whereas in other patients the lung disease might represent a reaction to a number of inhaled antigens, none of which appears to be exclusively responsible for the disease. Anyway, estimated prevalence varies by region, climate, and farming practices. In the US HP accounted for less than 2% of the patients with interstitial lung disease (ILD), whose yearly incidence was calculated to be about 30 per 100,000 [5]. A study carried out in Wisconsin on 1400 individuals estimated HP prevalence at 4.2% [6], while other data estimates HP affecting from 0.5 to 19.0% of exposed farmers [7]. In Europe, according to ILD registries, HP affects from 4 to 15% of all ILD cases [8], even if disease prevalence widely varies in different countries and within the same country due to geographic, seasonal, and climatic factors [9]. In a study of 431 incident cases in central Denmark, HP was the third most common ILD (7%), after idiopathic pulmonary fibrosis (28%) and connective tissue diseases (14%) [10]. In a Brazilian database including 3168 cases of ILD, prevalence of HP was 15%, the second place after connective tissue diseases 17% [11].

## Causative agents

Hypersensitivity pneumonitis can be caused by multiple agents that are present in work places and in the home, such as microbes, animal and plant proteins, organic and inorganic chemicals (Table 1). The number of environment, settings and causative agents is increasing over time. A relatively recent example are metal workers exposed to aerosolized metal working liquid often containing mycobacteria [12]. Studying an outbreak of metal fluid HP in an automobile manufacturing plant in Ontario, Canadian researchers found that when mycobacteria are established in the biofilms of the fluid system it is very difficult, if not impossible, to eradicate them [13]. HP has been observed also in wood transformation plants [14], peat moss packaging units [15] and in saxophone player, in this case caused by molds that grow inside the instrument [16]. These isolated cases underline the importance of a carefully taken environmental history in presence of symptoms suggesting the presence of an HP. On the other side, even in the context of a single specific exposure, there can be several potential antigens that might trigger the inflammatory pulmonary response [17]. One example of this complexity is the bird-fancier’s disease, in which the causative bird-related antigens include immunoglobulins, intestinal mucin present in bird droppings or blooms, a waxy substance coating the feathers of birds [18]. Another example is the complex ecosystem that could be observed in a contaminated humidifier system, such as *Klebsiella* species bacteria and Pullularia, Aureobasidium, Curvularia, Chaetomium, Penicillium, and Cephalosporium molds. Small amounts of these antigens also can be present in lake water, soil, and outdoor air in summertime [19]. The continuous research of etiologic agents in occupational and non occupational exposures is of outstanding importance because an extensive knowledge of these causative substances not only allows a better diagnosis and treatment but also leads to a reduction of the exposure.

**Table 1:** Hypersensitivity pneumonitis causative antigens

Antigen	Source	Disease
Microbes		
*Alternaria* species	Wood or wood pulp	Woodworker’s lung
*Aspergillus clavatus*	Moldy grains	Malt-worker’s lung
*Aspergillus* species	Tobacco mold	Tobacco-worker’s lung
*Aspergillus* species	Moldy malt	Malt-worker’s lung
*Aspergillus versicolor*	Animal bedding	Dog house disease
*Aureobasidium pullulans*	Moldy sequoia dust	Sequoiosis
*Aureobasidium* species	Contaminated water	Sauna-taker’s disease
*Bacillus subtilis*	Detergent enzymes	Detergent-worker’s lung
*Botrytis cinerea*	Grape mold	Winegrower’s lung or Späetlase lung
*Candida albicans*	Saxophone mouthpiece	Sax lung
Cephalosporium	Sewage	Sewage-worker’s lung
*Cryptostroma corticale*	Moldy maple bark	Maple bark–stripper’s lung
*Merulius lacrymans*	–	Dry rot lung
Mixed amoeba, fungi, and bacteria	Cold mist and other humidifiers, air conditioners	Nylon plant or office worker’s or air conditioner’s lung, ventilation pneumonitis
*Mycobacterium avium*	Contaminated water	Hot tub lung
*Mycobacterium* species, Gram negative bacilli	Metal-cutting fluid	Machine-worker’s lung
*Mucor stolonifer*	Paprika	Paprika-splitter’s lung
*Penicillium casei*	Cheese mold	Cheese-washer’s lung
*Penicillium chrysogenum*	Moldy wood dust	Woodworker’s lung
*Penicillium frequentans*	Moldy cork	Suberosis
*Saccharopolyspora rectivirgula* (micropolyspora faeni)	Moldy hay	Farmer’s lung
*Thermoactinomyces vulgaris*	Moldy hay, compost	Farmer’s lung, mushroom-worker’s lung, composter’s lung
*Thermoactinomyces sacchari*	Sugar cane residue	Bagassosis
* Thermophilic actinomycetes*	Moldy plant materials	Farmer’s lung
*Trichosporon* cutaneum	Mold in Japanese homes	Summer-type HP
Animals		
Animal fur protein	Animal fur	Furrier’s lung
Avian proteins	Bird excreta, blood, or feather	Bird-breeder’s lung, bird-fancier’s lung, pigeon-breeder’s lung
Gerbil proteins	Gerbil	Gerbil-keeper’s lung
Fish	Fish meal dust	Fishmeal-worker’s lung
Mollusk shell protein	Mollusk shell dust	Oyster shell lung
Ox and pork protein	Pituitary snuff	Pituitary snuff–taker’s lung
Rat proteins	Rat urine or serum	Rodent-handler’s lung
Silk worm larvae proteins	Silk worm larvae	Sericulturist’s lung
Wheat weevil	Flour	Miller’s lung
Plants		
Coffee	Coffee bean dust	Coffee-worker’s lung
Lycoperdon species	Puffballs	Lycoperdonosis
Soybean	Soybean hulls	Soybean-worker’s lung
Chemicals		
Anhydrides	Plastics	Chemical-worker’s lung, plasticworker’s lung, epoxy-worker’s lung
Bordeaux mixture	Vineyard fungicide	Vineyard-sprayer’s lung
Isocyanates	Paints, plastics	Paint-refinisher’s lung
Pauli’s reagent	–	Pauli’s reagent lung
Pyrethrum	Insecticides	Insecticide lung
Metals		
Cobalt	–	Hard metal lung disease
Beryllium	–	Berylliosis

Derived and adapted from Costabel et al [20]

## Pathogenesis

Inhaled antigens less than 5 µm in diameter may reach the lung parenchyma, moving to the lymphatic vessels and depositing at the level of respiratory bronchioles. Pathogens involved in HP cause similar clinical features, with an almost exclusive involvement of distal airways, alveolar and interstitial infiltration by inflammatory cells, and high titers of serum precipitating antibodies against the antigens responsible of the alveolar inflammation (precipitins), with normal levels of IgE and eosinophils. The pathogenesis of HP is quite similar regardless of the causative agent: the inflammatory response of the alveolar mucosa is a hypersensitivity reaction of type 3 (immune-complex-mediated) or type 4 (T lymphocytes mediated) [21]. The pathology is characterized by a bronchiolocentric interstitial mononuclear cell infiltration, small non-necrotizing epithelioid cell granulomas poorly formed, diffuse cellular pneumonitis and variable degrees of pulmonary fibrosis. Granulomas were commonly observed in the bronchiolar wall and alveolar ducts in subacute hypersensitivity pneumonitis; they are less than 150 µm in diameter, smaller than those observed in sarcoidosis [22]. Precipitins against causative antigen and immunoglobulin and complement were demonstrated in vessel walls [23]. T-lymphocytes mediated hypersensitivity response is the most important type 4 immune reaction in the pathogenesis of HP. Th1-cytokine network plays a key role in the development of HP [24], and later in the chronic form develops a Th2-like immune response. In fact, the features associated with chronic HP include a gradual increase in CD4+ T cells and in the CD4+/CD8+ ratio, a modification toward TH2 T cell differentiation and cytokine profile as well as a decline of CD8+ T cells [25]. In acute HP, pulmonary parenchyma inflammation appears to be mainly mediated by a type 3 response, as suggested by the presence of high titers of antigen-specific precipitating IgG in the serum, and an increase in lung neutrophils. Subacute and chronic forms of HP are characterized by a T cell-mediated immune response with increased T-cell migration and developing of a characteristic T-lymphocytic alveolitis [25]. The genetic factors determining individual predisposition to HP are still unclear, but in pigeon breeders disease (PBD) patients Camarena et al. [26] showed a significant increase of the alleles HLA-DRB1*1305 and HLA-DQB1*0501, while a decrease of HLA-DRB1*0802 was noticed in patients versus both control groups. Moreover, PBD patients had an increased frequency of TNF-2(-)(308) compared with both control groups. This subpopulation of patients exhibiting the TNF-2(-)(308) allele were younger, and displayed more lymphocytes in their bronchoalveolar lavages. These results suggest that genetic factors located within the MHC region contribute to the development of PBD.

## Clinical presentation

From the clinical point of view HP can be can be divided in acute/subacute, and chronic phenotypes, depending on the intensity and frequency of exposure to causative antigens. See Table 2 for classification and diagnostic criteria.

**Table 2:** Classification and diagnostic criteria of hypersensitivity pneumonitis

Characteristics	Acute/subacute HP	Chronic HP
Exposure to causal antigen	Intermittent high-level exposure	Continuous low-level exposure
Onset of symptoms	2–9 h after exposure; may evolve to gradually increasing symptoms over days to weeks	Insidious, over weeks to months
Nature of symptoms	Cough and dyspnea, but predominantly influenza-like symptoms	Progressive symptoms (dyspnea, cough, and weight loss), sometimes punctuated by intermittent attacks of symptoms or slowly increasing
Physical signs	Fever	Inspiratory crackles; cyanosis; digital clubbing; cor pulmonale
Outcome	Symptoms peak within 6–24 h after exposure, lasting hours to days. Symptoms recur on re-exposure and may progress to severe dyspnea	End-stage fibrotic disease and/or emphysema. Exacerbations may occur despite avoidance of exposure

Derived and adapted from the cluster analysis of the HP Study Group [3]

The acute form occurs after hours or days of antigen exposure, that generally is a short-term and intermittent exposure. The patient’s symptoms begin with fever, cough, dyspnea, asthenia and malaise that may persist for about a week after the causative agent exposure ends. The exacerbations might coincide with returning to work and subside when the subject is away from the working environment or from the allergenic setting for a sufficient period. On the chest auscultation could be noted widespread crackles in both chest walls, but sometimes auscultation is negative. The radiologic manifestations of acute HP are those of acute pulmonary edema, a high-resolution CT could be performed to better evaluate these patients [27]. The high-resolution CT scans could show patchy or diffuse bilateral ground-glass opacities and, especially in subacute forms, poorly defined small centrilobular nodules and lobular areas of decreased attenuation. The ground-glass opacities primarily reflect a diffuse lymphocytic interstitial pneumonitis, while the poorly defined centrilobular nodules may be caused by cellular bronchiolitis or focal areas of organizing pneumonia. The lobular areas of decreased attenuation and air trapping are presumably caused by small-airway obstruction by cellular bronchiolitis or, less commonly, by constrictive bronchiolitis [28]. During the acute episode respiratory function is reduced, also with DLCO alteration. Blood tests may show a slight eosinophilia with normal levels of IgE.

Subacute HP, a phenotype particularly difficult to identify, as demonstrated by the cluster analysis of 168 patients with HP performed by the international HP Study Group [3], is caused by a more prolonged exposure to the agent compared with the acute form. The onset is clinically sneaky, with productive cough, dyspnea, asthenia. For the rest, the subacute form is not particularly different than the acute form and the classification of HP should be restricted to two phenotypes, as suggested by the recent EAACI Position Paper on Occupational HP [29]. Chronic HP occurs as consequence of a continuous exposure to the pathogen, which causes a constant inflammation eliciting over time an irreversible pulmonary fibrosis. The chronic form has a more insidious onset than the subacute one, developing over a period of months or years, with progressive dyspnea with episodes of wheezing and recurrent low-grade fever. Symptoms, followed over time by a respiratory failure often evolve to pulmonary fibrosis [11]. Chest X-ray shows a diffuse interstitial fibrosis; spirometry demonstrates a restrictive syndrome, sometimes with obstructive patterns. DLCO is very low. In the chronic form, several months of low-level exposure to the offending allergen can result in very insidious respiratory symptoms with dyspnea, cough, sometimes mucopurulent sputum, anorexia and weight loss. The pattern of the chronic form can be similar to other forms of fibrotic pulmonary disease, evolving in most cases towards a progressive and irreversible disease despite avoidance of exposure to the causative agent and steroid treatment.

## Diagnosis

HP is often unrecognized and frequently misdiagnosed as respiratory infection or idiopathic interstitial lung disease because of its relatively low incidence in the general population. Moreover, the wide variability in clinical presentation of the disease HP often leads to the risk of underdiagnosis, unless the condition is specifically considered as a diagnostic possibility. In some patients, respiratory symptoms are the main feature of the clinical presentation, whereas in others people, systemic manifestations such as anorexia and weight loss overshadow other symptoms that, when present, might be intermittent, corresponding to episodic exposures to the causative agent, or might be always present, as in chronic form of the disease.

A high index of suspicion and a careful history, conducted in a systematic manner to insure that all relevant environmental and occupational information are obtained, are the main keys to make a correct diagnosis of HP. Once the suspicion of HP arises on the basis of initial history-taking, the diagnostic procedure could aim not only to identify the causative agent, but also to establish a temporal relationship between environmental exposures and initial onset of symptoms as well as temporary clinical manifestations. The diagnosis is often suspected when there is evidence of a respiratory disease with a history of relevant environmental or occupational setting and a clear relationship between symptoms and exposure. Physical examination and routine laboratory tests are generally not helpful, with total IgG often elevated, rheumatoid factor often positive, and peripheral eosinophil count generally normal as well as serum IgE levels. The suspected causative agent could be identified with the identification of specific precipitating antibodies in the patient’s serum, although specific precipitins can be found in many, but not all, patients with HP because of the low sensitivity due to poorly purified antigens, because of failure to adequately concentrate the patient’s serum prior to testing or, finally, because of the lack of the causative agent in the test panel [3, 30]. Moreover, it should be underlined that from 40 to 50% of asymptomatic individuals exposed to the same causative antigen have specific serum IgG antibodies [31]. In patients with a suspect of HP, a careful environmental and occupational history should guide the use of specific panels of precipitating antibodies, but the lack of consensus on disease definition limits the possibility to reach a certain diagnosis, although many diagnostic criteria for HP have been proposed [3]. Aiming at validating clinical diagnostic criteria for HP, in 2003 the HP study group analyzed a prospective cohort of consecutive patients with HP, identifying six significant predicting factors: (1) exposure to a known causative antigen, (2) presence of precipitins to the suspected causative antigen, (3) recurrent respiratory and systemic symptoms, (4) inspiratory crackles detected by physical examination, (5) symptoms occurring 4–8 h after exposure, (6) weight loss [27]. Pulmonary function tests can be normal in acute HP, while in chronic forms they typically demonstrate a restrictive pattern with small lung volumes and decreased (DLCO), although none of these features are specific to HP. Chest X-ray may provide some information regarding the extent of lung involvement: patients with acute or subacute HP may show bilateral interstitial and alveolar nodular infiltrates that could be patchy or homogeneous, while in chronic disease are present diffuse reticulonodular infiltrates and fibrosis, and honeycombing may occur. However, chest X-ray alone is not diagnostic, and a normal chest X-ray does not exclude the presence of HP. Instead of chest X-ray, high resolution CT (HRCT) scan has become important in the diagnosis of HP. In subacute form HRCT shows lobular areas of decreased attenuation or air-trapping, patchy or diffuse ground-glass opacities, and small centrilobular nodules [32]. In chronic HP the most common findings are traction bronchiectasis, interlobular septal thickening, and intralobular reticulation, with a distribution mainly peribronchovascular with no areas of predominance [33]. Other diagnostic procedures can be helpful in the diagnosis of HP, such as bronchoalveolar lavage (BAL) fluid analysis and lung biopsies. The presence of a lymphocyte count greater than or equal to 25% in BAL suggests a granulomatous disease such as sarcoidosis or HP, while a lymphocyte count greater than 50% is suggestive of HP, especially when associated with a neutrophils count greater than 3% and a mast cell count greater than 1% [34]. The utility of transbronchial biopsies (TBB) is limited by non specific results obtained in up to 48% of samples, however the presence of some findings such as diffuse lymphocytic infiltrates can be strongly suggestive for HP [35]. Surgical lung biopsies are more sensitive than TBB, and in subacute stages of disease the histologic findings can show the presence of interstitial lymphoplasmocytic pneumonitis, giant cells or non-necrotizing granulomas and cellular bronchiolitis. In the chronic stages of HP a predominantly fibrotic pattern mimics other types of interstitial lung disease particularly usual interstitial pneumonia (UIP), and a certain diagnosis can be always supported by clinical and radiological findings [36]. In conclusion, no gold standard for diagnosis of HP is currently available. Occupational or environmental exposure to a known causative agent, recurrent symptoms after exposure, inspiratory crackles, positivity of precipitating antibodies, and eventually weight loss were indicative findings of HP, together with BAL, HRCT, and, if needed, other diagnostic procedures such as lung biopsy.

## Treatment

The mainstay in managing HP is the avoidance of the causative antigen, though the complete removal is not always possible due to the difficulties to identify the agent or because its avoidance may lead to major changes in life style or occupational settings [37]. Some studies have suggested that HP could be not always a progressive disease, even if the job was not changed [38], suggesting a complex interaction between environmental and genetic factors [39]. If the allergen avoidance is not feasible or does not result in a complete symptoms relief, corticosteroid therapy is indicated. Corticosteroids may be useful either in relieving acute symptoms, or in subacute and chronic forms of HP, but they do not appear to have any effect on the long-term outcome of disease [40]. Anyway, a reasonable scheme therapy is oral prednisone between 40 and 60 mg, or equivalent doses for other corticosteroids, administered for a few days to 2 weeks in acute HP or for 4–8 weeks in subacute/chronic forms, followed by a gradual tapering to a maintenance dose of approximately 10 mg/day, or, if the patient clinical response is particularly good, to the discontinuation of the corticosteroid treatment. However, it should be emphasized that the efficacy of corticosteroid treatment lasting 12 weeks is not significantly superior to that of 4 weeks’ duration [41]. Interestingly, recurrence of acute farmer’s lung was more frequent among patients treated with corticosteroids who had also a prolonged antigen exposure, raising the hypothesis that steroid therapy was also suppressing the counter regulatory immune response. Depending on the patient clinical features, some supportive therapies may be prescribed, such as oxygen therapy if blood oxygen saturation is permanently under 90%, bronchodilators to open the airways, and opioids to control shortness of breath or chronic cough that is resistant to other treatments. If opioids are used several times a day, for several weeks or longer, it can lead to physical dependence and possibly addiction. In some cases the chronic form could not respond to corticosteroid treatment, evolving toward a progressive pulmonary fibrosis; in this eventuality lung transplantation should be considered, but it is important to remember that the procedure is not a cure: if after transplantation the patient will be exposed again to the causative antigen, a new allergic inflammation may severely damage again also the transplanted lung. Treatment is more successful when HP is diagnosed in the early stages of the disease, before permanent irreversible lung damage has occurred.

## Prognosis

Even though mortality trends are poor in the literature, in England and Wales from 1968 to 2008 878 deaths due to HP were described, with a mortality rate higher in men than women and with increasing age [42]. In a Danish cohort selected from high-quality registry, which provides real-life data with a diagnosis based on the current diagnostic criteria, the 5-year survival in the HP group was 93% [43]. Depending on the causative antigen, some studies suggested that bird-fancier’s hypersensitivity pneumonitis might have a worse prognosis than farmer’s lung, probably because patients with fibrosis on HRCT and/or lung biopsy have a poorer prognosis [44]. Factors associated with worse prognosis include an exposure for a longer period [45], older age, a histologic pattern of either fibrotic NSIP or UIP [46], digital clubbing [47], and a greater intensity of exposure [3].

## Conclusions

HP is a complex syndrome that needs urgently for more stringent and selective diagnostic criteria and validation, including wider panels of IgG, and a closer collaboration with occupational physicians, as part of a multidisciplinary expertise. The occupational HP was recently defined by the EAACI Position Paper on Occupational HP [29], and the definition proposed may partly be applied to the proportion of cases of non-occupational HP. The disease has not only protean clinical manifestations, but also a substantial overlap with other ILDs, and its pathogenesis is not fully understood. Further research is warranted to develop prognostic markers that can drive clinical decision making, even to establish worldwide registers and multicenter networks including tissue and imaging repository, to increase our knowledge on evolution of the different disease forms. Corticosteroids may improve symptoms in the short term, but additional studies are needed to test their long term effects and other immunosuppressive drugs in HP patients. Finally, there is no evidence on the efficacy of anti-fibrotic agents such as nintedanib and pirfenidone in the treatment of chronic HP [48], making it necessary further research.
